# Production of Low-Potassium Content Melon Through Hydroponic Nutrient Management Using Perlite Substrate

**DOI:** 10.3389/fpls.2018.01382

**Published:** 2018-09-19

**Authors:** Md. Asaduzzaman, Md. Raihan Talukder, Hideyuki Tanaka, Makoto Ueno, Mikiko Kawaguchi, Shozo Yano, Takuya Ban, Toshiki Asao

**Affiliations:** ^1^Olericulture Division, Horticulture Research Centre, Bangladesh Agricultural Research Institute, Gazipur, Bangladesh; ^2^Department of Agriculture, Faculty of Life and Environmental Science, Shimane University, Matsue, Japan; ^3^Department of Environmental Science, Bangabandhu Sheikh Mujibur Rahman Agricultural University, Gazipur, Bangladesh; ^4^Faculty of Home Economics, Otsuma Women’s University, Tokyo, Japan; ^5^Faculty of Medicine, Shimane University, Izumo, Japan; ^6^Faculty of Agriculture, Tokyo University of Agriculture and Technology, Fuchu, Japan

**Keywords:** melon, potassium restriction, low-potassium melon, soilless culture, perlite substrate, chronic kidney disease

## Abstract

Chronic kidney disease patients are restricted to foods with high potassium content but our daily diets including melon are rich in potassium. Therefore, we investigated the production of low-potassium melon through hydroponic nutrient management in soilless culture using perlite substrate during autumn season of 2012, 2014 and spring season of 2016. In the first study, melon plants were supplied with 50% standard ‘Enshi’ nutrient solution until first 2 weeks of culture. In 3rd and 4th week, amount of applied potassium was 50, 75, 100, and 125% of required potassium nitrate for each plant per week (based on our previous study). It was found that, melon plants grown with 50% of its required potassium nitrate produced fruits with about 53% low-potassium compared to control. In the following study, four cultivars viz. Panna, Miyabi shunjuukei, Miyabi akifuyu412, and Miyabi soushun banshun309 were evaluated for their relative suitability of low-potassium melon production. Results showed insignificant difference in fruit potassium content among the cultivars used. Source of potassium fertilizer as potassium nitrate and potassium sulfate and their restriction (from 1 or 2 weeks after anthesis) were also studied. There were no influences on fruit potassium content and yield due to sources of potassium fertilizer and restriction timings. In our previous studies, it was evident that potassium can be translocated from leaves to fruits at maturity when it was supplied nutrient without potassium. Thus, we also studied total number of leaves per plant (23, 24, 25, 26, and 27 leaves per plant). It was evident that fruit potassium, yield, and quality were not influenced significantly due to differences in number of leaves per plant. These studies showed that restriction of potassium nitrate in the culture solution from anthesis to harvest could produce melon fruits with low-potassium (>20%) content compared to potassium content of greenhouse grown melon (340 mg/100 g FW). Quality testing and clinical validation of low-potassium melon also showed positive responses compared to greenhouse grown melon.

## Introduction

Potassium plays important role in human body and maintains normal functioning of muscles, heart, and nerves through acid base equilibrium, enzymatic activation, and renal function (Russell, 2009; Crawford and Harris, 2011). It acts as the main electrolytes, largely accumulated within body cells and usually excreted through kidneys. However, patients with kidney dysfunctions can’t excrete it completely and thus concentrated in blood outside cells. This increased levels of potassium inside human body cause hyperkalemia (Kes, 2001), which is a common life threating issue for the chronic kidney disease (CKD) patients (Jain et al., 2012). They are advised to avoid foods with higher potassium content. However, our daily dietary items including melon are rich in potassium (Weiner and Wingo, 1998). This food restriction becomes more severe in case of CKD patient taking dialysis. They are also suggested not to take raw vegetables with high potassium content. It is reported that before eating, excessive potassium in these vegetables can be partially removed by cutting into smaller pieces, boiled or soaked sufficiently in water (Burrowes and Ramer, 2008). Vegetables preparation following above methods may also result in loss of other nutrients and water soluble vitamins and minerals, and breakdown of desirable texture and taste as reported in lettuce (Yakushiji and Kagawa, 1975; Kimura and Itokawa, 1990). Therefore, production and supplementation of fruits and vegetables containing lower potassium would greatly improve dietary components of CKD patients.

Recently, hydroponic production technologies of low-potassium fruits and vegetables such as melon, strawberry, tomato, spinach, and lettuce have been developed in Japan (Ogawa et al., 2007; Asao, 2011; Mondal et al., 2016; Tsukagoshi et al., 2016). In general, quantitative management of nutrient solution was applied to restrict potassium nutrition without hampering plants normal growth and development. Plants were grown with standard nutrient solution during the early growth stage and then the culture solutions were replaced either by a non-potassium hydroponic fertilizer or supplied nutrient solution without potassium fertilizer at the later growth stage. Following this method potassium content of fruits and vegetables can be reduced without hampering normal growth and development of plants. This type of cultivation method is now commercially applied by Aizufujikako Co., Ltd. (Tokyo, Japan) to develop low-potassium content leaf lettuce. Hydroponic nutrient solution contains sufficient amount of essential nutrients and plant roots can uptake them luxuriously. If it is applied continuously, plants can uptake essential ions at very low concentrations. In a study, no adverse effect on growth, fruit yield, and fruit quality in tomato was reported when there is reduction of macronutrient concentrations to 50% of the control level (Siddiqi et al., 1998). High levels of potassium in the nutrient solution were found to increase fruit dry matter, total soluble solid content, and lycopene concentration of tomato (Fanasca et al., 2006) and strawberry (Ebrahimi et al., 2012; Rodas et al., 2013). Source of potassium fertilizer as potassium sulfate had influence on yield, and quality of passion fruit (Costa-Araujo et al., 2006) and strawberry (Khayyat et al., 2007) while potassium nitrate found to have no influence in pepper (Flores et al., 2004).

According to a dietitian, dialysis patients can eat melon if it contains 40% reduced potassium of a general melon (Standard Tables of Food Composition in Japan, 2011). In this regard, our research group aimed at developing low-potassium melon fruits with reduced potassium compared to general melon. In previous studies, we succeeded in reducing the potassium concentration in melon fruit (40% or more) by limiting the potassium nitrate concentration in the culture solution from the vegetative growth stage till harvest in container based hydroponics (Asao et al., 2013). When melon plants were grown with 1/4 potassium nitrate of standard nutrient solution, fruits potassium decreased to about 39% compared to control. In the following study, it was found that melon plants cultured with 1/16 and 0 levels of potassium nitrate produced fruits with 35 and 43% reduced potassium, respectively, compared to control. However, stable production of melon fruits with reduced potassium under large scale and using commercial soilless substrate would be useful and practical.

In solution culture, excessive potassium absorption and its accumulation in plant parts and remobilization to edible sink have been reported (Asao et al., 2013). Thus, it is difficult to find out the minimal potassium requirement to the plants. It was evident that, if potassium supply is restricted to zero then growth hampers, fruit quality decreases and fruit cracking occur. In this regards, use of suitable soilless media can provide ideal conditions of potassium absorption and increase both yield and quality crops. Rockwool has been widely used as growing media for horticultural crops because it has stable structure, high water holding capacity, and moderate porosity (Sonneveld, 1993; Smith, 1998; Raviv and Lieth, 2008; Asaduzzaman et al., 2012). However, this inorganic substrate is difficult to degrade, and waste often stockpiled resulting potential environmental risk (Cheng et al., 2011). Recently, perlite has emerged as an excellent growth medium for growing several horticultural crops including melon (Szmidt et al., 1988; Cantliffe et al., 2003; Hochmuth and Hochmuth, 2003; Fascella and Zizzo, 2005; Rodriguez et al., 2006). Its strong capillary attraction draws up solution from the bottom of substrate bag or container at the similar rate that the plants uptake water and nutrient leaving excess solution in the reservoir. Therefore, it enables optimum moisture conditions near root while in case of rockwool it is difficult to maintain optimum moisture levels because of its poor capillary action. In Florida, Holland, and United States, melons are being grown using 100% perlite substrate in bags or in containers. It can be recycled and reused for several years after cleaning and disinfecting properly (Hanna, 2005, 2006, 2010). Therefore, we used perlite substrate in potassium nutrient management studies for producing low-potassium melon.

We investigated four independent studies aiming stable production of low-potassium melons and their dietary supplementation to CKD patients. In this regard, several nutrient and cultural management practices were investigated to develop sustainable low-potassium melon production technology. Melon plants were grown in perlite substrate supplied with several percentages of required potassium during vegetative growth period. Effect of varietal differences, timing of potassium restriction, and number of remaining leaves after pinching on growth, fruit quality, and potassium content of melons were studied.

## Materials and Methods

### Melon Cultivar

Five cultivars of melon (*Cucumis melo* L. cv. Panna, Miyabi shunjuukei, Miyabi akifuyu412, Miyabi soushun banshun309 and Miyabi natsu206) were used for this study. These netted melons were grown in greenhouse following standing culture supported with jute ropes. One fruit per plant was maintained throughout the studies. The seeds of these melon cultivars were collected from Takii & Co., Ltd., Kyoto, and Yokohama Uki Co., Ltd., Tokyo, Japan. The cultivars have excellent sweetness with either green or orange flesh.

### Enshi Nutrient Solution

Melon plants were grown in soilless culture using perlite bags with 50% ‘Enshi’ nutrient solution which is generally recommended for melon cultivation in Japan with an electrical conductivity (EC) of 1.32 dS/m and pH of 6.93 (Hori, 1966; **Supplementary Table S1**). In this present study, we have reduced the amount of potassium nitrate keeping other nutrients constant to produce low-potassium melon fruit in soilless culture. The slightly higher pH (6.93) of this nutrient solution, it is assumed to be contributed by the higher pH of tap water (pH 7.93) of laboratory that might lead to higher pH range of the nutrient solution used. It is mentionable that source of tap water in our research facility is the mountain nearby. The inherent nutritional composition of tap water used was also not so great but calcium, magnesium, and nitrate-nitrogen content were little higher (**Supplementary Table S2**). In our previous cultures and also in this present experiment, we did not find any nutritional deficiency in melon culture with pH value of 6.93.

### Experimental Conditions

Four independent experiments were carried out in 100 m^2^ glasshouses and 100 m^2^ plastic house of Experimental Research Center for Biological Resources Science, Shimane University in hydroponics and soilless culture using perlite substrate. In the greenhouse, we used the only the central portion with plant density of 60 plants while in the plastic house 175 plants were accommodated in five different rows. The studies were conducted during the autumn season 2012, 2014 and spring season of 2016. The study area is generally characterized by a moderate weather condition. During the culture period of 2012, 2014, and 2016 the mean day/night temperatures were 26.9/20.0, 26.0/19.6, 25.1/19.4°C, respectively (**Supplementary Figure S1**).

### Experiment I: Quantitative Management of Nutrient Solution of Melon Using Perlite Substrate

Seeds of melon cv. Panna were sown in cell trays with vermiculite. Germinated seedlings with high vigor and uniform in size were transplanted into plastic container with 50% ‘Enshi’ nutrient solution for nursery. After 1 week at five to seven leave stage, one seedling was planted in one plastic container filled with 30 L perlite substrate (**Supplementary Figure S2A**). For the first 2 weeks, melon plants were supplied with 50% standard ‘Enshi’ nutrient solution. In 3rd and 4th weeks, standard nutrient solutions were supplied in four splits viz. 50, 75, 100, and 125% of required potassium nitrate per plant (**Supplementary Table S3**). In our previous study, we calculated the amount of potassium nitrate required for one plant per week in hydroponic culture of melon (Asao et al., 2013). Female flowers of 11–14 nodes were kept for fruit development and others were removed. At these nodes, female flowers of secondary branches on first collateral node were pollinated and the branches were punched leaving the second node, and main shoot tips were punched at 25th node. After pollination, melon plants were supplied with standard nutrient solution without potassium nitrate. Melon plants grown with 50% standard nutrient solution in perlite and also in hydroponics were used as control.

### Experiment II: Varietal Difference in Low-Potassium Melon Cultivation

Seeds of four melon cultivars viz. Panna, Miyabi shunjuukei, Miyabi akifuyu412, Miyabi soushun banshun309 were sown in cell tray with vermiculite on July 24, 2014. After germination, seedlings were transferred into plastic container with 50% ‘Enshi’ nutrient solution for nursery on August 31. Then similar size and vigor seedlings were transferred to plastic bag filled with 10 L perlite on August 11 (**Supplementary Figure S2B**). One plant was planted per bag for each variety having three replications and five bags in each replication. The plants were supplied with 50% standard nutrient solution. On the bottom of the perlite bag, a hole was made for discharging the surplus supplied nutrient solution. Potassium nitrate in the culture solution was supplied according to the growth of plants and the total supply amount became 30940 ml until 4 weeks then potassium nitrite supply was stopped (**Supplementary Table S4**). Pollination started on September 2 and female flowers of 11–14 nodes were kept for fruit development and others were removed. At these nodes, female flowers of secondary branches on first collateral node were pollinated and the branches were punched leaving the second node, and main shoot tips were pinched at 25th node on September 15. The cultivar “Panna” was harvested on October 29 and “Miyabi” cultivars were harvested on November 3.

### Experiment III: Effects of Timing of Potassium Fertilizer and Potassium Deficiency on Melon Growth and Fruit Quality

Seeds of melon cv. Miyabi natsu206 were sown in cell trays with vermiculite on April 4, 2016. After germination, the similar sized seedlings with good growth were selected and transferred to plastic pots with perlite for raising seedlings as nursery on April 19. Then selected healthy plants were planted into plastic bag filled with 10 L of perlite on May 3. After planting at 5–6 leaf stage, the plants were supported with rope to standing upright with iron wire (**Supplementary Figure S2C**). All the lateral shoots were removed except 12–14 nodes for flowering. Both male and female flowers booming started on June 3 and pollination of the flowers from 12 to 14 was done manually. After 7–10 days of pollination when size of the fruit was about the size of Ping-Pong balls, fruit thinning was performed leaving only one fruit per plant on June 13. In order to study the potassium content per fruit, we maintained one fruit per plant for all the cultures. At the same time, main shoot tips were punched at the 25th node. There were two timings of potassium fertilizer restriction for this experiment viz. 1 week after anthesis (June 11) and 2 weeks after anthesis (June 18). There were two sources of potassium fertilizer such as potassium nitrate and potassium sulfate. Melon plants were supplied with 50% standard nutrient solution from seedling planting to plant growth at 15th leaf stage. From May 28, plants were supplied with 75% nutrient solution (**Supplementary Table S5**).

### Experiment IV: Influence of Different Number of Remaining Leaves on the Growth and Fruit Quality of Melons After Punching

Seeds of melon cv. Miyabi natsu206 were sown in cell trays with vermiculite on March, 2016. After germination, the seedlings of the same size with good growth were selected. On March 17, the seedlings were transferred to the seedling raising pot with perlite. A hole was drilled in a culture bag filled with 10 L of perlite and seedlings were planted on April 11. After transplanting, plants at 5–6 leaf stage were supported by jute rope to iron wire (**Supplementary Figure S2C**). All the lateral shoots were removed except 12–14 nodes for flowering. Both male and female flowers booming started on May 19 and pollination of the flowers from 12 to 14 was done manually. After 7–10 days of pollination when the size of the fruit was about the size of Ping-Pong balls, fruit thinning was performed leaving only one fruit per plant. At the same time, main shoot tips were pinched at the nodes leaving 23–27 leaves on June 3. Potassium nitrate was used as the potassium fertilizer for this culture. The timing of limiting potassium nitrate was 1 week after anthesis on May 16. From transplanting to the 17th leaf stage, melon plants were supplied with 50% standard nutrient solution. From May 14, the plants were supplied with 75% standard nutrient solution (**Supplementary Table S6**).

### Analysis of Melon Fruit Qualities

Melon fruits were harvested and stored for 4 days (cv. Panna) and 7 days (cv. Miyabi) at room temperature for maturation. After maturation, melon fruits were cut into small pieces, mixed by juicer (Zojirushi BM-RS08-GA, Zojirushi Corporation, China) and then mixed juice was used for analyzing soluble solids, titratable citric acidity, and ascorbic acid. Soluble solid contents in melon sample were measured using a pocket digital refractometer (PAL-1, Atago Ltd., Tokyo, Japan) and repeated measurements were conducted after washing the prism with distilled water and also rinsed with the test juice. For determination of titratable acidity, 2 ml of melon juice were poured into a conical flask with 8 ml of distilled water and then 2 drops of phenolphthalein were added. At measurement, pH was adjusted to 8.2 using 0.1 N (w/v) NaOH. The quantity of NaOH (ml) required, and the amount for appropriate acidity was converted to citric acidity (%). Ascorbic acid contents in melon samples were determined following 2,4-dinitrophenylhydrazine (DNP) colorimetry. In 50 ml glass test tube, 0.5 ml of melon juice were taken and then 0.5 ml of 10% meta-phosphoric acid solution, 1 ml of distilled water, 1 ml of 0.03% 2,6-dichlorophenol–indophenol (DCP), 2 ml of thiourea, and 1 ml of DNP were added. The samples were then incubated for 3 h at 37°C in water bath (BW400, Yamato Scientific Co., Ltd., Japan). After incubation 5 ml of 85% H_2_SO_4_ were added keeping the test tubes cold water with ice bag. After cooling for about 30 min, ascorbic acid content was measured at 520 nm by Spectrophotometer (U-2900, Hitachi High Technologies Corporation, Tokyo, Japan).

### Determination of Potassium and Other Mineral Content in Melon Fruit

The concentration of potassium, calcium, magnesium, iron, and sodium in melon fruits was measured using polarized Zeeman Atomic Absorption Spectrophotometer (Z-2310, Hitachi High Technologies Corporation, Tokyo, Japan). After maturation, edible parts of melon fruits (about 10 g) were measured by analytical Balance, XS204DRV, Greifensee, Switzerland) and placed in a 250 ml plastic bottle that contains 200 ml of 1% HCl. Then the samples were shacked in a bio shaker (Bio-Shaker BR-43FL, Japan) for 30 min at 150 rpm for complete extraction of mineral nutrients. Fruit samples were then filtrated (Advantec Grade No. 131) and analyzed for mineral contents.

### Determination of Potassium and Other Mineral Content in Melon Plant Parts

Melon plant parts were divided into leaves, stem, and roots (only for hydroponic culture) and dried in a constant temperature oven (DKN 812, Yamato Scientific Co. Ltd., Japan) at 80°C for at least 72 h. Then plant parts were ground with a mixer machine (National MX-X53, Japan). Powdered samples (0.5 g) were mixed with 8 ml of HNO_3_ and digested using microwave sample preparation system (ETHOS1, Milestone S.r.l, Bergamo, Italy). The digested samples were measured up to 50 ml of volumetric flask with distilled water and then filtered through qualitative filter paper (Grade no. 131). The filtered sample solutions were analyzed for potassium, calcium, magnesium, iron, and sodium by Zeeman Atomic Absorption Spectrophotometer.

### Statistical Analysis

Analysis of variance was performed to test for significant effects of different potassium nitrate levels in the nutrient solution, different variety, potassium fertilizers and time of restriction, and number of remaining leaves after pinching on the plant growth variables, fresh weight per fruit and qualities and mineral nutrients in plant parts of melon in all four studies. Mean separations were performed by Tukey–Kramer test (Statcel 2 Software, OMS publication, Tokorozawa, Saitama, Japan) at *P* < 0.05.

## Results and Discussion

Potassium is an essential mineral nutrient and it has major physiological role in normal growth and development of plants (Taiz and Zeiger, 1998; Schachtman and Liu, 1999; Mengel, 2007; Szczerba et al., 2009). Thus, its requirement and uptake by plant roots are also high (Tisdale and Nelson, 1975; Mäser et al., 2002; Britto and Kronzucker, 2008; Szczerba et al., 2009). Hydroponic nutrients contain sufficient amount of all essential minerals for plants luxurious uptake. However, plants can uptake essential nutrients even at very low concentrations from a standard concentration (Hoagland and Arnon, 1950; Hori, 1966). Therefore, higher concentrations of nutrients are either not used by plants or their uptake does not influence higher production (Zheng et al., 2005; Rouphael et al., 2008). In this study, we limited the potassium fertilizer in hydroponic culture solution and investigate its impact on the fruit potassium content of melon. Four independent melon cultures were carried out in both hydroponics and soilless culture during both spring and autumn seasons from 2012 to 2016. In hydroponic culture technique, nutrient management is easy, simple and accurate. Nutrient concentration and composition of the culture medium can be modified during any growth stages of plant. In our studies, we measured growth parameters, dry matter partitioning and yield of melon at different potassium management strategies. Mineral nutrient content including potassium was also investigated to understand the source-sink relations due to potassium restriction in the culture solution.

### Quantitative Management of Nutrient Solution of Melon Using Perlite Substrate

In our previous study, we determined the amount of weekly potassium absorption per melon plant in order to apply potassium nutrition in a quantitative manner (Asao et al., 2013). It was found that potassium requirement was great until 4th weeks of planting and then the demand decreased sharply. The absorption of potassium per plant was 11, 20, 33, 76, and 69 ppm in 1st, 2nd, 3rd, 4th, and 5th week of vegetative growth, respectively. In addition, plants grown in standard nutrient solution absorbed a higher amount of potassium whereas plants grown in nutrient solution with decreased potassium levels absorbed potassium proportionate to the applied amount in culture solution. The above study concludes that potassium requirement of melon plants remain greater at vegetative growth stage before anthesis and then decreases gradually and during fruit development absorption turns to minimum. As the tap water used for this study contains low concentration of potassium (about 1.0 ppm), even if we grow plant in solution lacking potassium fertilizer, still it can absorb potassium at minimum levels throughout the growing period (**Supplementary Table S2**). Based on these results we applied potassium at different percentages of absorbed potassium per plant in a week in order to investigate the lowest limit of potassium requirement.

#### Plant Growth and Chlorophyll Content

Plant growth variable such as stem length, dry weight of leaves and stem, and photosynthetic parameters were significantly influenced by the potassium nitrate management in perlite substrate and hydroponics culture (**Table 1**). In general, stem length was found shorter in perlite culture compared to hydroponics. In hydroponics, plants absorbed mineral nutrient luxuriously leading larger plant body. On the other hand in perlite culture, plant roots move toward the required nutrients. Stem lengths from 1–11 nodes (S1) and 12–25 nodes (S2) were significantly similar in all levels of potassium nitrate applied. Dry weights of leaves and stem were significantly higher in plants grown in hydroponics than in perlite culture. Plants grown in perlite using different levels of potassium nitrate produced significantly similar dry weight except for leaves from 12–25 nodes (L2) in 50- and 75% potassium application per plant. Relatively higher chlorophyll content was recorded in leaves in melon plants grown in hydroponics compared to perlite culture in both leaf positions (leaves at 15 and 20 nodes). In case of perlite culture, significantly lower leaf chlorophyll content was found in plant supplied with 50% of potassium nitrate. Dry matter production in plants grown in perlite was decreased to about 30–50% compared to plants grown in hydroponics. Similarly, it was reported that limited supply of potassium in the culture solution can inhibit growth and performance of tomato plants (Kanai et al., 2007).

**Table 1:** Effect of limited supply of potassium in nutrient solution on the growth, chlorophyll content, yield and fruit qualities in leaves of melon grown in soilless culture using perlite substrate.

KNO_3_ supply	Length (cm)		Dry weight (g)				SPAD		Fresh weight/fruit (g)	Soluble solids content (%)	Titratable citric acidity (%)
						
	S1	S2	L1	L2	S1	S2	L15	L20			
Hydroponics (50% std.)	103.7 a^y^	105.7 a	45.7 a	41.4 a	11.0 a	10.0 a	53.8 a	57.0 a	2410.4 a	14.7	0.20
Perlites (50% std.)	101.0 ab	98.5 ab	23.1 b	28.9 b	7.0 b	5.7 b	38.3 b	42.3 b	1750.4 b	15.5	0.19
50%^z^	98.8 ab	95.1 b	22.3 b	23.0 c	7.6 b	6.0 b	29.4 c	28.6 c	1647.3 b	14.0	0.15
75%	100.9 ab	98.9 ab	22.3 b	25.1 bc	7.1 b	5.8 b	34.4 bc	31.6 bc	1674.8 b	14.5	0.18
100%	95.4 b	97.0 b	22.7 b	27.4 b	7.7 b	6.3 b	33.4 bc	37.5 bc	1681.4 b	15.3	0.17
125%	101.2 ab	97.9 b	22.2 b	27.3 b	8.0 b	6.4 b	31.4 bc	35.0 bc	1724.4 b	15.3	0.17
Significance										ns	ns


**^*z*^Percentage of potassium absorption per plant. ^*y*^Means within a column followed by different letters are significantly different and “ns” indicates non-significant according to the Tukey’s test at P < 0.05*. *L1, leaves from 1 to 11 nodes; L2, leaves from 12 to 25 nodes; S1, stem length from 1 to 11 nodes; S2, stem length from 12 to 25 leaves; L15, SPAD taken at leaf of 15 node; L20, SPAD taken at leaf of 20 node*.*

#### Fruit Yield and Quality

**FIGURE 1:**
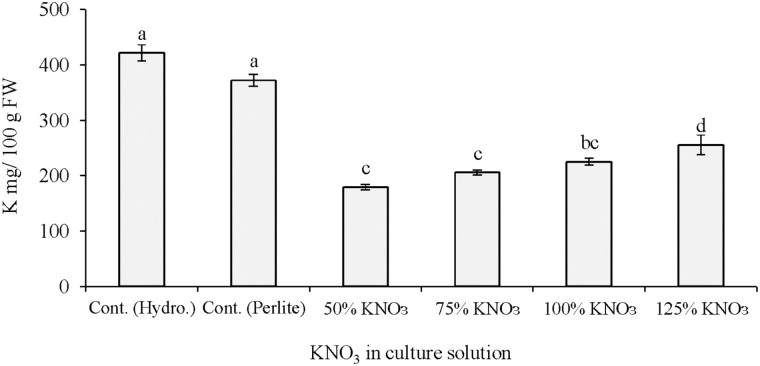
Effect of limited supply of potassium fertilizer in the nutrient solution on the potassium content of melon fruits. Significant differences among treatments are indicated by different letters at *P* < 0.05. Error bars shows SE (*n* = 11). In perlite culture, 50–125% KNO_3_ indicates percentage of potassium absorption per plant.

It is reported that adequate amount of potassium is necessary for improving yields and fruit qualities such as fruit size and color, soluble solids and ascorbic acid content, shelf life and shipping quality of several horticultural crops (Geraldson, 1985; Lester et al., 2005, 2006, 2007; Kanai et al., 2007). In the present study, comparatively higher fruit yield was recorded in plant grown in hydroponics with 50% standard nutrient solution than perlite culture with 50% standard nutrient solution or its limited levels (**Table 1**). This yield reduction is mainly due to system differences of hydroponic solution culture and soilless culture using perlite substrate. It is evident from the results that fruit yield did not differ in the reduced potassium levels (50–125% of required potassium) and standard potassium nutrition. Fruit yield is supposed to decrease under potassium restricted cultivation. However, in our previous study fruit yield also not decreased due to potassium restriction in hydroponic solution (Asao et al., 2013). This indicates that the reduced levels of potassium fertilizer in the nutrient solution can still maintain potassium sufficiency for melon in hydroponics. Fruit qualities such as soluble solids and citric acid content did not differ significantly among the nutrient solutions applied either in hydroponics or in perlite culture. Plant cultivation under potassium limited condition needs special cultural practices. Simple reduction of potassium in the culture medium may not reduce the potassium in the fruits or edible parts. In this regards, the quantitative management of potassium in the nutrient solution during vegetative growth and restricted supply in the reproductive stage may result in fruits with lower potassium content. In the present study, potassium content in melon fruits was significantly influenced by the application of different types of nutrient solution in hydroponics and perlite culture (**Figure 1**). In general, plants grown with 50% standard nutrients showed higher fruit potassium in both culture systems. In case of perlite culture, plants grown with 50% of required potassium nitrate during 3 and 4th weeks produced fruits with the lowest potassium (53%) compared to perlite control. Under this potassium nitrate treatment, fruit potassium decreased to about 47% (179.4 mg/100 g FW) compared to potassium content in greenhouse melon (340 mg/100 g FW; Standard Tables of Food Composition in Japan, 2011). Similar results were found in our previous study, where about 39–43% fruit potassium was decreased due to the reduction in potassium nitrate fertilizer from 1/16th to 0 levels of standard hydroponic solution (Asao et al., 2013).

#### Fruit Mineral Nutrient Content

**FIGURE 2:**
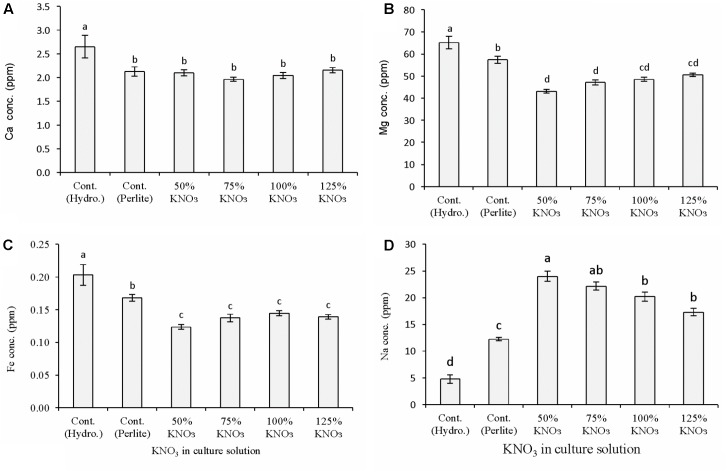
Effect of limited supply of potassium fertilizer in the nutrient solution on the mineral nutrient content of melon fruits. Sub-figures **(A–D)** referring to Ca, Mg, Fe, and Na, respectively. Significant differences among treatments are indicated by different letters at *P* < 0.05. Error bars shows SE (*n* = 11). In perlite culture, 50–125% KNO_3_ indicates percentage of potassium absorption per plant.

Potassium nitrate fertilization significantly affected the macro- and micronutrient content of fruits in both hydroponics and perlite culture (**Figure 2**). In general, calcium, magnesium, and iron concentration in hydroponic melon fruit was higher than the potassium nitrate levels in perlite culture (**Figures 2A–C**) while only sodium concentration showed the opposite results. Calcium concentration was similar in all the potassium nitrate levels. In perlite culture, magnesium concentration was decreased significantly in reduced potassium nitrate levels than 50% standard nutrient solution especially in 50 and 75%. Iron concentration was also showed a similar trend of decrease in reduced potassium nitrate levels than standard nutrient solution. Sodium concentration showed antagonistic trend than other minerals, especially potassium content in fruits (**Figure 1**) due to application of different types of nutrient solution (**Figure 2D**). Fruit sodium content was significantly decreased in hydroponics culture with 50% standard nutrient solution followed by perlite control. While in perlite culture, sodium content increase with the decrease of potassium nitrate concentrate in the culture solution and it was greater in 50% potassium nitrate level compared to other levels.

In the first experiment, melon plants grown in hydroponics and in perlite with reduced potassium nitrate affected the mineral nutrient content in the fruits. In general, the concentration of calcium, magnesium, and iron was decreased compared to control plants in hydroponics and perlite culture. This is due to the minimal supply of potassium nitrate in the perlite substrate. Sodium concentration in fruits showed clear antagonistic interaction with the potassium availability in the culture solution (**Figures 1**, **2D**). In case of limited supply of potassium nitrate, sodium concentration increase with the decrease of required potassium nitrate concentration in perlite culture. Compared to control plants in hydroponics, about 80 and 60% increased sodium were measured in fruits from plants cultured in perlite with 50% of required potassium and with standard nutrient solution, respectively. In other studies, potassium restriction in the culture solution showed significant increase in sodium and magnesium concentrations of leafy vegetables and tomato (Ogawa et al., 2012). Increased concentration of magnesium and sodium were reported to be compensated for the reduction of potassium. It was reported that availability of sodium can lead to up to 95% of the maximum yield at the critical level of potassium in field vegetable crops (Greenwood and Stone, 1998). In another study, it was found that decreased levels of potassium can increase sodium and magnesium content in tomato (Diem and Godbold, 1993; Pujos and Morard, 1997). Thus, supply of these two minerals should be considered in the studies dealing with potassium deficiency.

#### Mineral Nutrient Content in Plant Parts

**FIGURE 3:**
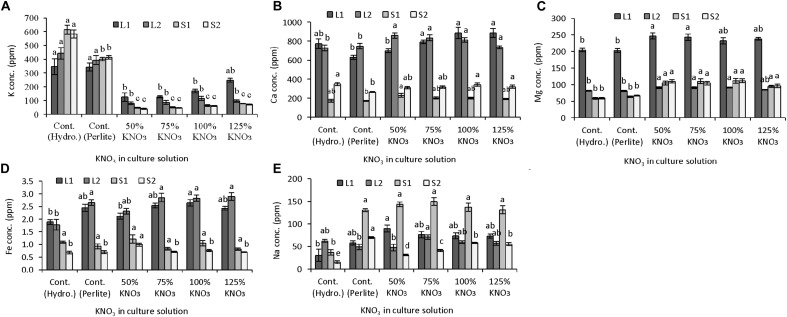
Effect of limited supply of potassium fertilizer in the nutrient solution on the mineral nutrient content in plant parts of melon. Sub-figures **(A–E)** referring to K, Ca, Mg, Fe, and Na concentration in LI (1–11 nodes), L2 (12–25 nodes), SI (1–11 nodes), and S2 (12–25 nodes), respectively. Significant differences among treatments are indicated by different letters at *P* < 0.05. Error bars shows SE (*n* = 11). In perlite culture, 50–125% KNO_3_ indicates percentage of potassium absorption per plant.

Potassium nitrate restriction affected the micro- and macronutrient partitioning in leaves and stem of plants grown in hydroponics and perlite substrate (**Figures 3A–E**). Potassium content was mostly affected by fertilizer treatment. In leaves, potassium content decreased greatly (up to 64%) in limited potassium supply compared to plants grown with 50% standard nutrient solution in hydroponics and perlite substrate (**Figure 3A**). Both lower leaves (from 1 to 11 nodes) and upper leaves (12–25 nodes) showed a similar pattern of potassium content and it decreased with the decreased concentration of potassium nitrate in nutrient solution. In case of potassium content in stem, it was also decreased with the limited supply of potassium nitrate in the nutrient solution (**Figure 3A**). When 50% standard nutrient solution was supplied, stem potassium in plants grown in hydroponics was significantly higher than the plants grown in perlite substrate. Moreover, in perlite culture, potassium content was greatly reduced in S1 (81–88%) and S2 (83–90%) of plants grown with limited potassium nitrate (75–125%) compared to standard nutrient solution. It also revealed that potassium content in stem parts (S1 and S2) was significantly similar in limited potassium nitrate levels.

Accumulation of mineral nutrient was studied in parts of leaves and stem in order to reveal their accumulation and translocation under limited potassium condition. It was found that calcium and iron content was not decreased but was similar or higher in leaves and stems of plants grown in reduced potassium concentrations. In case of magnesium, an antagonistic interaction was observed in all parts (L1, L2, S1, and S2). Its concentration was increased in all levels of reduced potassium than that of control either in hydroponics or in perlite. Unlike fruit sodium content, its concentration in different parts of leaves and stem is increased significantly in plants supplied with reduced levels of potassium compared to hydroponics with standard nutrient solution. Antagonistic relations of potassium and sodium have been reported in faba bean and tomato as well (Cordovilla et al., 1995; Song and Fujiyama, 1996). It also reported that antagonistic interaction of sodium with calcium, potassium, and zinc showed adverse effects in plant growth (Shukla and Mukhi, 1979) on the other hand, it can replace osmotic function of potassium in plants and show position effect (Marschner, 1995; Subbarao et al., 2003; Ali et al., 2006; Kronzucker et al., 2008; Kronzucker and Britto, 2011).

### Experiment II: Varietal Difference in Low-Potassium Melon Cultivation

#### Plant Growth

Plant growth variables and dry weights in plant parts significantly differed among cultivars grown under limited potassium nitrate cultivation using perlite substrate (**Table 2**). Plant height was significantly shorter in cultivar “Panna” than three “Miyabi” cultivars. Among the “Miyabi” cultivars “Miyabi akifuyu412” produced the tallest plant. Although maximum leaf length was not varied, comparatively wider leaves were produced by “Miyabi” cultivars than “Panna.” While three “Miyabi” cultivars produced significantly similar wider leaves. Dry weight of leaves under the fruiting nodes (L1) and also the above nodes (L2) were not significantly differed among the cultivars grown in limited potassium cultivation. In case of stem dry weight, all three “Miyabi” cultivars produced higher dry weight than “Panna.” “Miyabi shunjuukei” produced lower S2 dry weight compared to other two “Miyabi” cultivars. In cultivation method of low-potassium leafy vegetables for dialysis patients, it was observed that plant growth in nutrient solution with lower potassium concentration had no significant influence on growth compared to the control plants (Ogawa, 2018).

**Table 2:** Growth parameters, yield and fruit quality of four melon cultivars grown in perlite substrate with limited potassium supply.

Cultivars	Plant height (cm)	Maximum leaf length (cm)	Maximum leaf width (cm)	Dry weight (g)				Fresh weight/fruit (g)	Soluble solids (%)	Titratable citric acidity (%)	Ascorbic acid (ppm)	Cracked fruits/15 plants
									
				L1	L2	S1	S2					
Panna	117.6 c^z^	16.1	21.0 b	7.3	23.0	3.3 b	4.5 c	1142.6 b	10.0	0.4 b	309.9 b	7
Miyabi shunjuukei	158.1 b	15.3	23.1 ab	7.2	25.2	4.7 a	5.1 bc	1115.8 b	10.6	0.6 ab	490.3 a	8
Miyabi akifuyu412	188.9 a	15.9	27.1 a	7.1	24.2	5.0 a	6.0 a	1364.7 a	9.1	0.7 a	416.5 a	2
Miyabi soushun banshun309	165.4 b	15.1	22.9 ab	7.1	24.3	4.5 a	5.6 ab	1152.4 b	9.8	0.6 a	472.3 a	3
		ns		ns	ns				ns			


**^*z*^Means within a column followed by different letters are significantly different and “ns” indicates non-significant according to the Tukey’s test at P < 0.05. L1, leaves from 1 to 10 nodes; L2, leaves from 11 to 25 nodes; S1, stem length from 1 to 10 nodes; S2, stem length from 11 to 25 leaves.**

#### Fruit Yield and Quality

Fruit yield under restricted potassium cultivation may vary due to growing seasons and environmental conditions. It has also been reported that potassium uptake depends on plant factors, including genetics (cultivars) and developmental stage such as vegetative versus reproductive stages (Rengel et al., 2008). Therefore, in the second study, we evaluated four melon cultivars for their potential mechanism of low-potassium fruit production. Results indicated that fruit yield and quality in four melon cultivars were influenced significantly under limited potassium nitrate fertilization in perlite culture (**Table 2**). The average fruit weight was significantly greater in “Miyabi akifuyu412” than other cultivars grown in this cultivation. The other two “Miyabi” cultivars including “Panna” produced significantly similar fruit weight. Soluble solid content was varied significantly among the cultivars and overall sweetness (about 10% soluble solids) was not so high compared to generally grown greenhouse melon (about 13% soluble solids). Tritratable citric acidity was lower in “Panna” compared to “Miyabi” cultivars while there were no significant differences among three “Miyabi” cultivars. Similar results were observed in case of ascorbic acid. Ascorbic acid content was found higher in three “Miyabi” cultivars than “Panna” under limited potassium nitrate application in perlite culture. In our low-potassium study with strawberry, we found that fruit qualities were affected by the strawberry cultivars in addition to reduced level of potassium nitrate (Mondal et al., 2016). Among the cultivars and potassium nitrate level, “Toyonoka” with 1/1 level of potassium nitrate produced higher ascorbic acid contents strawberry fruits while it was lower in other cultivars with 1/16 of potassium nitrate. Research results revealed that there were no differences in the other mineral contents between low-potassium lettuce and normal leaf lettuce, except for higher sodium and lower nitrate contents in low-potassium lettuce (Yoshida et al., 2014). Fruit cracking was observed and recorded in four melon cultivars grown under limited potassium nitrate cultivation (**Table 2**). Regarding the number of cracked fruit, comparatively fewer cracked fruits were recorded in “Miyabi akifuyu412” (2) followed “Miyabi soushun banshun309” (3). While greater number of cracked fruits were harvested from “Miyabi shunjuukei” (8) and “Panna” (7).

#### Fruit Mineral Content

**FIGURE 4:**
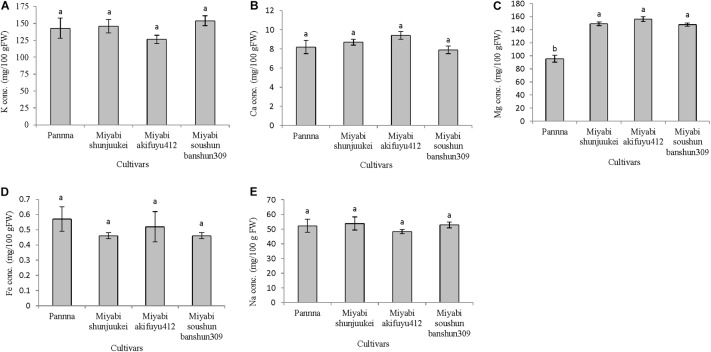
Mineral nutrient content in fruits of four melon cultivars grown under limited potassium supply in nutrient solution. Sub-figures **(A–E)** referring to K, Ca, Mg, Fe, and Na concentration in fruits, respectively. Significant differences among treatments are indicated by different letters at *P* < 0.05. Error bars shows SE (*n* = 10).

In the second experiment, all mineral nutrients except magnesium were not varied significantly among the four cultivars under limited potassium nitrate supply from anthesis (**Figures 4A–E**). The cultivar “Panna” has lower magnesium content in fruits compared to three cultivars of “Miyabi” series. In “Panna,” “Miyabi shunjuukei,” and “Miyabi soushun banshun309,” fruits potassium content was about 143–154 mg/100 g FW which is about 55–58% reduction compared to potassium content in generally grown greenhouse melon (340 mg/100 g FW; Standard Tables of Food Composition in Japan, 2011), while in “Miyabi akifuyu412” this reduction was the highest (63%) (**Figure 4A**). It is evident that the difference in uptake in potassium in “Panna” and “Miyabi” mainly was due to difference in genetics or species difference and also at different developmental stages of the plant (Rengel et al., 2008). Calcium content was similar in all four melon cultivars grown and range from 7.9 to 9.4 mg/100 g FW (**Figure 4B**). Magnesium content was significantly reduced in “Panna” compared to three “Miyabi” cultivars under limited potassium nitrate supply (**Figure 4C**). About 1.5 times greater than “Panna” but similar magnesium content (about 151 mg/100 g FW) was measured in three “Miyabi” cultivars. Iron content was not differed due to the limited supply of potassium nitrate in nutrient solution in four melon cultivars and it was ranged from 0.46 to 0.57 mg/100 g FW (**Figure 4D**). There were no significant differences in sodium content in fruits of four cultivars and it ranges from 48.3 to 53.8 mg/100 g FW (**Figure 4E**).

#### Mineral Nutrient Content in Plant Parts

**FIGURE 5:**
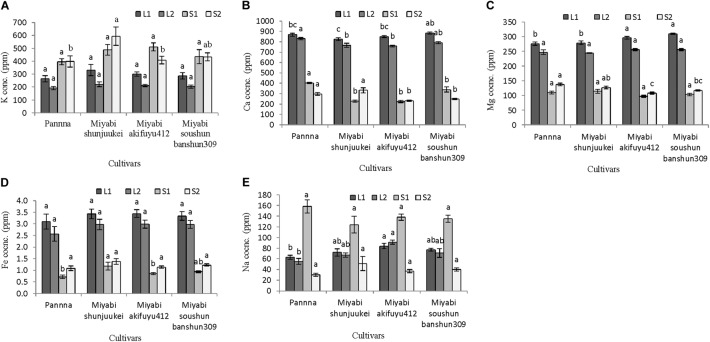
Mineral content in different plant parts of four melon cultivars grown under limited potassium supply in nutrient solution. Sub-figures **(A–E)** referring to K, Ca, Mg, Fe, and Na concentration in LI (1–10 nodes), L2 (11–25 nodes), S1 (1–10 nodes), and S2 (11–25 nodes), respectively. Significant differences among treatments are indicated by different letters at *P* < 0.05. Error bars shows SE (*n* = 10).

Potassium nitrate restriction in nutrient solution affected mineral nutrient content in leaves and stem of four melon cultivars (**Figures 5A–E**). In general, potassium content in stem was higher than leaves in all four cultivars (**Figure 5A**). In leaves either L1 or L2, potassium content did not differ significantly among the cultivars used. Compared to potassium content in plants grown with 50% standard nutrient solution (**Figure 3A**), about 3–23% potassium in L1 and 35–44% potassium in L2 were decreased in plants grown with limited potassium nitrate in four cultivars. Although potassium content in S1 was not different among the cultivars, its content was significantly affected in S2. In “Miyabi shunjuukei” potassium content of S1 part was significantly greater followed by “Miyabi soushun banshun309” and there was similar potassium content of S2 in other cultivars.

Calcium content showed opposite trend of potassium (**Figure 5B**), and in L1 of “Miyabi soushun banshun309” its content was highest compared to other cultivars while in L2, the same cultivar was followed by “Panna.” In case of calcium content in S1, the cultivar “Panna” was higher than that of three “Miyabi” cultivars and among “Miyabi” cultivars it was not varied significantly. However, in S2 calcium content was also higher in “Panna” followed by “Miyabi shunjuukei” and other two cultivars showed similar content. In general, magnesium content also higher in leaves than stems and significantly differed among cultivars used (**Figure 5C**). In L1, magnesium content was higher in “Miyabi akifuyu412” and “Miyabi soushun banshun309” compared to other two cultivars while it was not affected in L2. On the other hand, magnesium content in S1 did not differ but it was found higher in “Panna” and “Miyabi shunjuukei.” Iron content in leaves was much higher than in stem and it did not differ in L1, L2, and also S2 (**Figure 5D**). Its content in S1 was higher in “Miyabi shunjuukei” and “Miyabi soushun banshun309” compared to other two cultivars. Sodium content in different parts of leaves and stem were not varied in four cultivars used (**Figure 5E**). Interestingly, its content in S1 was about 2–5 times higher than S2. Potassium is known as the highly mobile mineral nutrient for plants. The above interaction might be due to the mechanism of cellular substitution of potassium by sodium related with osmotic balance and maintenance in cell vacuoles (Leigh, 2001; Amtmann et al., 2005; Ogawa and Yamauchi, 2006; Rengel et al., 2008).

### Effects of Potassium Fertilizer Source and Time of Restriction on Melon Growth and Fruit Quality

#### Fruit Yield, Quality, and Potassium Content

In the third study, source of potassium fertilizer and time of restriction had no significant influence on the fruit yield and potassium concentration (**Table 3**). However, soluble solid was affected due to potassium nitrate restriction and its interaction with source of fertilizer. This phenomenon indicates that potassium restriction after 1 week of anthesis would result in low-potassium fruits production. In this study, fruit potassium content was not decreased greatly compared to first and second studies and it is evident that about 25% potassium decreased compared to potassium concentration of Standard Tables of Food Composition in Japan (2011). Review of available researches showed that potassium fertilization had positive effects on the melon fruit yield, qualities, sensory attributes, and bioactive compound for human health (Lester et al., 2010). It was mentioned that these effects will depend on the mode of fertilization and source of potassium. While other studies found that potassium fertilization may have little or no influence on fruit qualities of cucumber, bell pepper, strawberry, and watermelon (Hochmuth et al., 1994; Albregts et al., 1996; Locascio and Hochmuth, 2002; Perkins-Veazie et al., 2003; Umamaheswarappa and Krishnappa, 2004). Other studies showed that, potassium nitrate applied either in soil or as foliar during middle to late season, there were little or no improvements of marketable yield and nutritional qualities in muskmelon (Jifon and Lester, 2009). In our previous study, the measured fruit qualities of melon were unaffected except citric acid content when reduced levels of potassium nitrate applied in hydroponics (Asao et al., 2013).

**Table 3:** Effect of different potassium fertilizer and potassium missing date on fruit weight, fruit potassium concentration and soluble solid content of melon cultivar grown in perlite bags.

Potassium fertilizer	Potassium missing date (month/day)^z^	Fresh weight/fruit (g)	Potassium conc. (mg/100g FW)	Soluble solid content (%)
KNO_3_	6/11	2027.6	256.6	11.5
	6/18	2091.6	258.0	10.8
K_2_SO_4_	6/11	1987.3	260.2	11.7
	6/18	2008.3	267.5	11.4
Analysis of variance	Potassium fertilizer	ns	ns	ns
	K missing date	ns	ns	∗
	Interaction	ns	ns	∗


**^*z*^Potassium fertilizer supply was stopped 1 week after anther (6/11) and 2 weeks after anthesis (6/18). “ns” indicates non-significant and asterisk (“∗”) indicates significant according to the Tukey’s test at P < 0.05.**

#### Dry Matter Partitioning in Plant Parts

**FIGURE 6:**
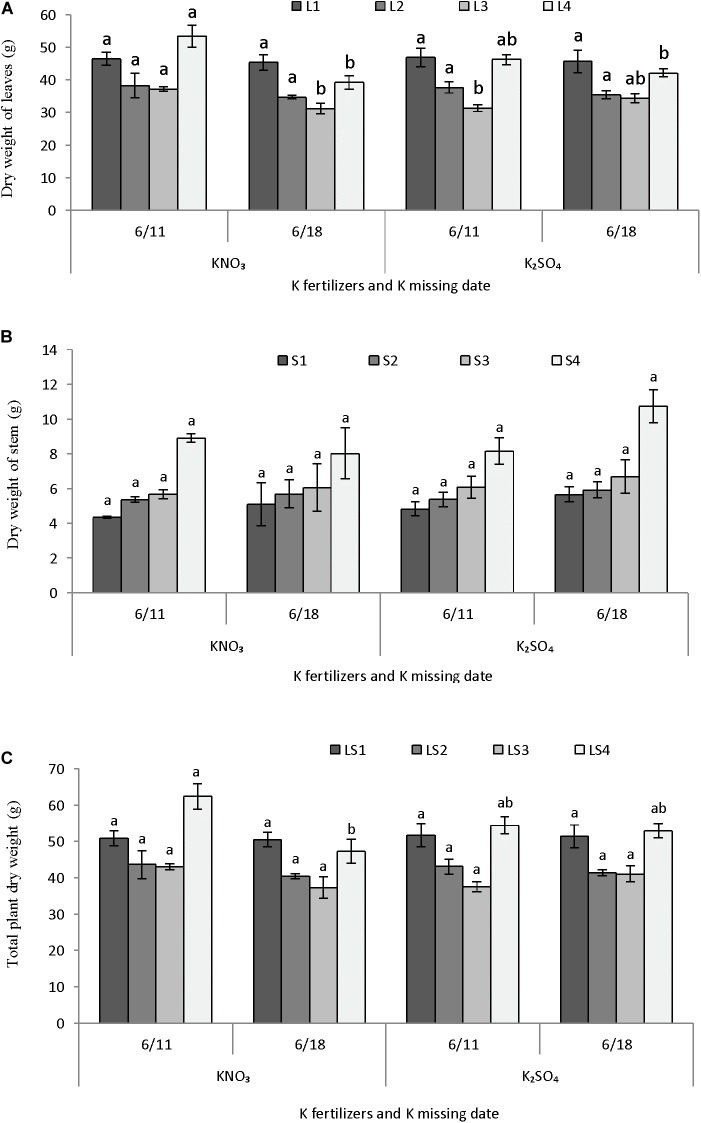
Effect of different K fertilizers and K missing dates on the dry matter production of melon. Sub-figures **(A–C)** referring to dry weight of leaves [LI (1–6 nodes), L2 (7–12 nodes), L3 (13–18 nodes), and L4 (19–25 nodes)], stem [S1 (1–6 nodes), S2 (7–12 nodes), S3 (13–18 nodes), and S4 (19–25 nodes)], and total plant [LSI (1–6 nodes), LS2 (7–12 nodes), LS3 (13–18 nodes), and LS4 (19–25 nodes)], respectively. Significant differences among treatments are indicated by different letters at *P* < 0.05. Error bars shows SE (*n* = 8).

Potassium fertilizers and time potassium restriction showed significant influence on the dry matter partitioning of melon in perlite substrate (**Figures 6A–C**). Dry weight of lower leaves L1 (1–6 nodes) and L2 (7–12 nodes) were not differed due to supply of either potassium nitrate or potassium sulfate and also potassium restriction after 1 or 2 weeks of anthesis (**Figure 6A**). The upper leaves L3 (13–18 nodes) had greater dry weight in potassium nitrate supply and potassium restriction 1 week after anthesis, which is similar to potassium sulfate supply with potassium restriction 2 weeks after anthesis. The uppermost leaves L4 (19–25 nodes) had higher dry weights when potassium restricted after 1 week of anthesis and applied either potassium nitrate or potassium sulfate. Dry weights in four parts of stem did not differ significantly due application of either potassium nitrate or potassium sulfate and potassium restriction times after anthesis (**Figure 6B**). In general, dry weight of stem parts showed an increasing trend from lower to upper part (S1–S4). Total plant dry weight as four different parts of leaf and stem (LS1–LS3) except the upper part (LS4) was also not differed significantly due to application of potassium fertilizers or potassium restriction times after anthesis (**Figure 6C**). Dry weight of LS4 was significantly higher in potassium nitrate supply and potassium restriction after 1 week of anthesis, which similar in case of potassium sulfate supply with potassium restriction either 1 or 2 weeks after anthesis.

#### Potassium Content in Plant Parts

**FIGURE 7:**
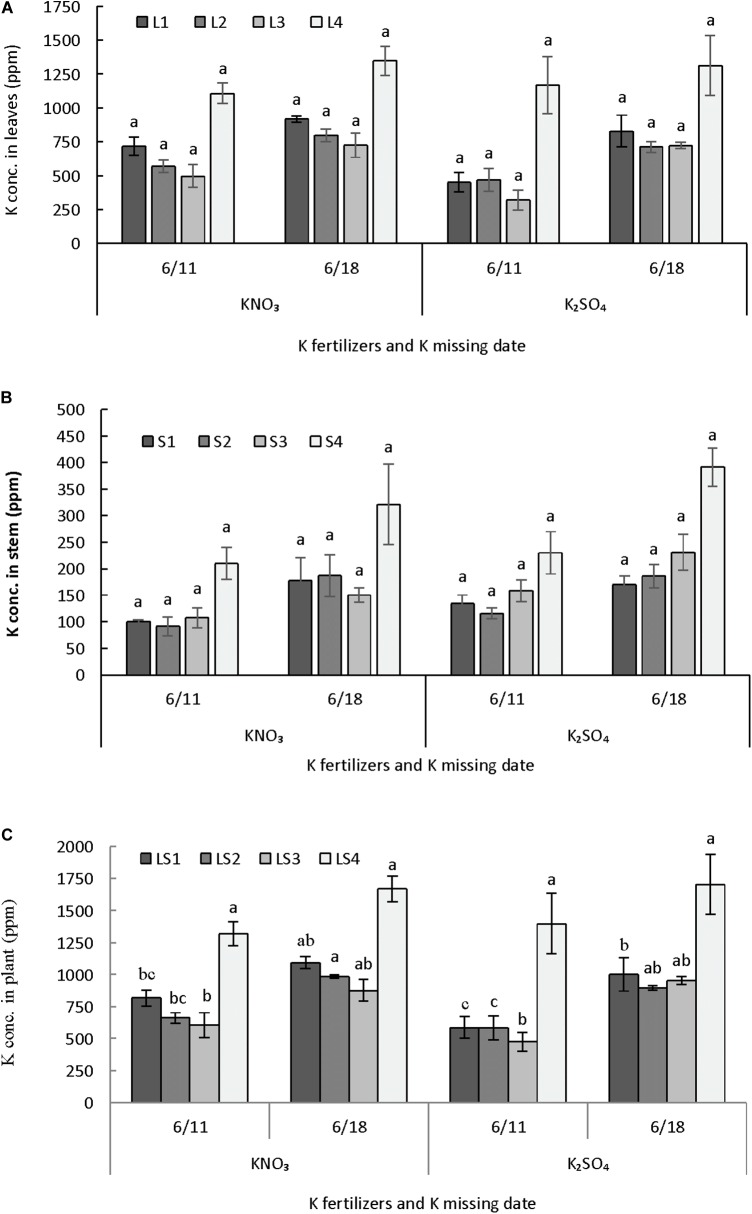
Effect of different K fertilizers and K missing dates on K concentration in different plant parts of melon. Sub-figures **(A–C)** referring to K conc. leaves [L1 (1–6 nodes), L2 (7–12 nodes), L3 (13–18 nodes), and L4 (19–25 nodes)], stem [S1 (1–6 nodes), S2 (7–12 nodes), S3 (13–18 nodes), and S4 (19–25 nodes)] and total plant [LS1 (1–6 nodes), LS2 (7–12 nodes), LS3 (13–18 nodes), and LS4 (19–25 nodes)], respectively. Significant differences among treatments are indicated by different letters at *P* < 0.05. Error bars shows SE (*n* = 8).

Potassium content in leaves and stem were not differed in parts due to application of potassium fertilizers and potassium restriction after anthesis but it was significantly different when leaves and stem considered together (**Figures 7A–C**). Potassium content was similar in leaves until 18 nodes (L1–L3) but it was higher in uppermost leaves (L4) (**Figure 7A**). Potassium content in stem followed the similar trend as it was observed in case of leaves (**Figure 7B**). Results revealed that potassium content in plant differed due to application of potassium nitrate or potassium sulfate and their restriction either 1 or 2 weeks after anthesis (**Figure 7C**). In lower portion of plant (LS1), potassium content was greater in plants grown with potassium restriction at 2 weeks after anthesis and supplied potassium nitrate fertilizer. In case of LS2 and LS3, potassium content was greater in plants with potassium restriction at 2 weeks after anthesis and application of either of the potassium fertilizers. LS4 was not affected by the application of two sources of potassium fertilizers and times of potassium restriction.

### Influence of Different Number of Remaining Leaves on the Growth and Fruit Quality of Melons After Pinching

#### Fruit Yield, Quality, and Potassium Content

Number of leaves remains in the plant can contribute yield and qualities of melon fruits. In this study, number of leaves per plant (23–27) has no significant influence on the fruit yield, potassium concentration, and soluble solid content (**Table 4**). The average fruit yield ranges from 1224.3 to 1578.2 g in plants with 23–27 leaves. Number of leaves per plant also had no significant influence on potassium content in fruits. The lowest potassium content was measured in fruits from plants with 23 leaves, which is about 27% lower than generally grown greenhouse melon (Standard Tables of Food Composition in Japan, 2011). Number of leaves indicate the photosynthetic site, which contributes the soluble solid content of fruits. However, it was found that soluble solid content did not differ due to variation of leaves form 23 to 27. Potassium as plant nutrient is required in large quantities, and its excessive deficiency would result in decrease in fruit quality (size, sugar content, etc.) through reduction of stomatal conductance and CO_2_ fixation (Cakmak, 2005). Thus, conversion of light energy to chemical energy impaired and ultimately phloem export of photosynthate from leaves to fruit decreased greatly. In this study, we examined whether low-potassium could be achieved without lowering fruit quality by adjusting the number of upper leaves (L4). It was shown that 23–25 leaves per melon plant are necessary for suitable fruit weight and optimum sugar content. The sugar content of fruits usually increased with the increase in number of remaining leaves, it increases the source of assimilate. Nishimura et al. (2008) recommended that at least 19 main leaves are necessary in order to maintain the quality of fruits in Earl’s melon cultivation. The increase in the number of leaves not only increases the production of photosynthetic products but also greatly influences the distribution of inorganic components to each organ and leaf, and it has a big influence on the fruit sugar content. In this study, it was considered 12 leaves above the fruit and the number of remaining leaves was 24 and fruit was in the later branch at 12 nodes position. It was thought that although supply and uptake of potassium from the culture solution is low but translocation from leaves and stem would be higher. From the above point, the number of remaining leaves after pinching is 24 was considered suitable for low-potassium cultivation. In order to increase the sugar content we increased the nutrient solution concentration from 50 to 75% in the experiments I and II and in turn fruit potassium content decreased only about 20%. However, results indicated that sugar was not increased due to increase in the nutrient solution concentration during fruit development. Thus, it is evident that 50% nutrient solution would be suitable for low-potassium melon cultivation from seedling raising to harvesting. In the present study, we also reduce the amount of nutrient supplied to plant before 2 weeks of harvest. This was done to increase the potassium deficiency which could produce low-potassium content melon through potassium-deficient gradient and concentration stress.

**Table 4:** Influence of differences in the number of leaves per plant on fruit weight, potassium concentration, and soluble solid content in melon.

Leaves per plant^z^	Fresh weight/fruit (g)	Potassium conc. (mg/100 g FW)	Soluble solid content (%)
23	1520.1	249.5	9.3
24	1578.2	276.5	9.9
25	1344.0	277.0	9.7
26	1224.3	301.9	9.9
27	1283.7	290.5	10.0
Significance	ns	ns	ns


**^*z*^Detopping was done leaving 23, 24, 25, 26, and 27 leaves from the base. “ns” indicates non-significant according to the Tukey’s test at P < 0.05*.*

### Quality Testing and Clinical Validation of Low-Potassium Melon

Low-potassium melon fruits produced in the above studies were tested for their quality response by high school students and also by dieticians (**Table 5**). Melon fruits with different concentration of potassium and soluble solid content received different responses. The highest score was received by the melon fruits with 148% potassium concentration followed by 25% potassium concentration. However, participant’s impression was that melon fruits with 148% potassium were bitter in taste while melon fruits with 25% potassium have less sweetness. Peoples generally like melon fruits with 51% potassium because it does not create stimulus inside the mouth. People with oral allergy syndrome like a comparatively low-potassium content melon.

**Table 5:** Serum potassium and sodium levels, blood pressure, and pulse before and after eating low-potassium melon (Adopted from Talukder et al., 2016).

Eating low-potassium melon	Serum potassium (mEq/L)	Serum sodium (mEq/L)	Systolic BP (mmHg)	Diastolic BP (mmHg)	Pulse (/min)
Before	4.6 ± 0.4	136.6 ± 3.1	118.7 ± 11.8	70.1 ± 11.4	71.6 ± 13.5
After	4.6 ± 0.3	137.7 ± 1.9	119.4 ± 14.8	67.8 ± 9.6	71.7 ± 15.1
*P*-value	0.5	0.67	0.58	0.92	0.39


**We served 100 g of low-potassium melon for dinner and measured serum potassium and sodium levels, blood pressure, and pulse in 9 CKD patients (estimated GFR <45 ml/min/1.73 m^*2*^) with mean age of 69 in a hospital. No significant change was determined before and 1 day after eating low-potassium melon in addition to their usual diet*.*

**FIGURE 8:**
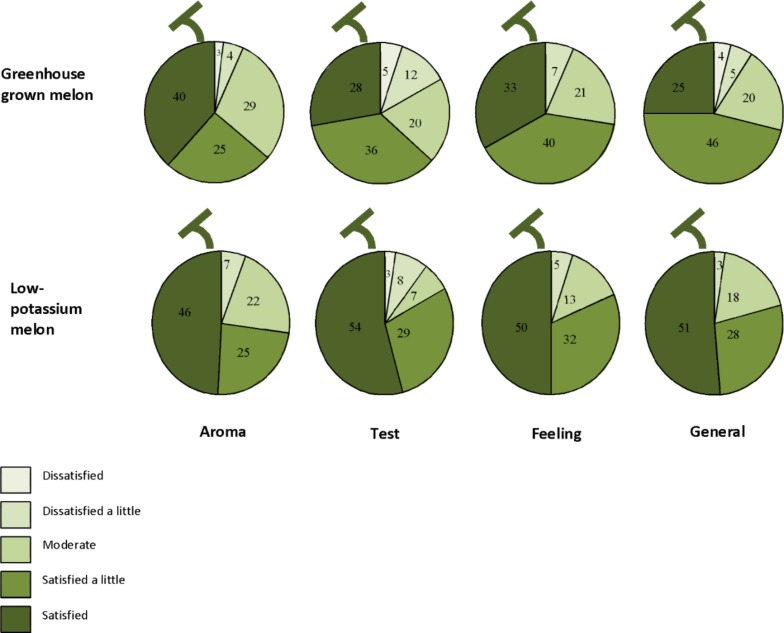
Results from a questionnaire regarding low-potassium melon in 76 dialysis patients (Adopted from Talukder et al., 2016).

We have tried to serve low-potassium melon for lunch or dinner in CKD patients to verify the safety (**Table 6**) and evaluate the effectiveness (**Figure 8**). We served 50 g of low-potassium melon and 50 g of normal melon blindly in 76 maintenance dialysis patients in their lunch box. After eating melon, they answered some questions regarding the aroma, taste, and feeling without any information about melon. Interestingly, they satisfied low-potassium melon at least as same as normal melon. Results were similar to those of healthy subjects (results not shown).

**Table 6:** Response of high school student and managerial dieticians after eating low-potassium content melons.

Potassium conc. in melon (%)	Soluble solid content (%)	High school students^z^	Managerial dieticians^y^	Mean value
148	12.9	5.0^x^	5.0	5.0
102	13.6	3.0	2.5	2.7
74	13.5	1.3	1.5	1.4
51	13.2	1.7	2.1	1.9
25	11.1	4.0	4.0	4.0


**^*z*^Figures are means of 9 high school students. ^*y*^Figures are means of 11 managerial dieticians from Shimane University medical hospital. ^*x*^Good taste score (high ∼ low) : 1 ∼ 5.**

Dietary potassium for human comes largely from fruits and vegetables. It is necessary for the normal water balance between the cells and body fluids. Studies indicate that the average daily potassium intake is 2000–3900 mg (Kes, 2001; Pollock et al., 2005; Choi and Ha, 2013), which is too high for patients with kidney dysfunction to excrete. Thus, potassium intakes are limited to <1500 mg/day (Stage 5 patients) or 2000–2500 mg/day (Stages 3 and 4 patients) for the CKD patients with hyperkalemia (Pollock et al., 2005). On the other hand, most of our dietary items such as fruits including melon, fresh vegetables, seaweed, beans, and potatoes contain high potassium. Therefore, dietary management is an important aspect of improvement for dialysis patients and also provides clinical guidelines that recommend intake of micronutrients (Kopple, 2011). Dietary supplementation also reported to prevent hyperphosphatemia, hyperkalemia, hypertension, and water retention (Mailloux, 2000; Heerspink et al., 2009; Kalantar-Zadeh et al., 2009; Sherman and Mehta, 2009; Noori et al., 2010; Sanghavi et al., 2013).

Therefore, we validated our results on low-potassium content melon by supplementing in diet of the patients suffering from CKD admitted at Shimane University Hospital. We served both melon fruits with higher potassium concentration (482 mg/100 g FW), and also lower potassium concentration (183 mg/100 g FW), to compare responses from dialysis patients in regard of sweetness, taste, and texture. It was observed that the majority of the patients responded for melon fruits having half concentration of potassium in Standard Tables of Food Composition in Japan. They also wanted to recommend this low-potassium melon to other patients. In addition, many of them mentioned that there was no tingling sensation of eating netted melon with low-potassium concentration. They also mentioned that it was delicious and easy to eat. Low-potassium melon, relieving the dietary restrictions of kidney disease patients, as there was no tingling sensation, everyone has a great feeling of eating such tasty melon.

We have also served low-potassium melon in the lunch or dinner menus of CKD patients to verify the safety and acceptability. We served 100 g of low-potassium melon for dinner and measured serum potassium and sodium levels, blood pressure, and pulse in 9 CKD patients (estimated GFR <45 ml/min/1.73 m^2^) with mean age of 69 in a hospital. There were no significant change determined before and 1 day after eating low-potassium melon in addition to their usual diet (**Table 5**). In addition, we also evaluated the sensory attributes after eating low-potassium melon compared to normal melon form 76 dialysis patients (**Figure 8**). We served 50 g of low-potassium melon and 50 g of normal melon blindly in 76 maintenance dialysis patients in their lunch box. After eating melon, they answered some questions regarding the aroma, taste, and feeling without any information about melon. Interestingly, they satisfied by low-potassium melon at least as same as normal melon. Results were similar to those of healthy subjects. We observed positive responses in favor of eating low-potassium melon. Better results would be observed through continuous effort toward low-potassium melon production technique. Although the current results are in the experimental stage, in order to realize the dissemination of this low-potassium melon technology, we would consider the further restriction of potassium from the vegetative growth stage of melon plants. In a recent study, the low-potassium lettuce evaluated as lower in bitterness but higher in saltiness compared to normal lettuce (Yoshida et al., 2014). The overall preference score and higher preference score were significantly higher for low-potassium lettuce. Therefore, low-potassium lettuce might be useful for improving the diets and other varieties of food for CKD patients suffering from hyperkalemia.

## Conclusion

In the first experiment, melon plants grown with reduced levels of potassium nitrate produced fruits with lower potassium content in perlite culture than in hydroponics. It was found that if 50% of required potassium nitrate supplied during 3rd and 4th weeks of after planting and then without potassium nutrition till harvest, fruit potassium decreased considerably (53%) compared to control. Under this quantitative potassium management, four cultivars were evaluated and there was no difference in fruit potassium content was evident but fruit potassium content was considerably lower than the general melon. In “Panna,” “Miyabi shunjuukei,” and “Miyabi soushun banshun309,” fruits potassium content was about 143–154 mg/100 g FW which is about 55–58% reduction compared to potassium content in generally grown greenhouse melon (340 mg/100 g FW), while in “Miyabi akifuyu412” this reduction was the highest (63%). In the third study, source of potassium fertilizer such as potassium nitrate and potassium sulfate and timing of potassium restriction form anthesis did not show any influence on the potassium content of melon. Through this study, compared to control at best 25% low-potassium in fruits was produced in plants grown with potassium nitrate and it restriction after 1 week of anthesis. Number of leaves per plant considered to be source of photosynthate and similarly source of potassium to be translocated during fruit development even under potassium restriction. However, our fourth study indicated that number of leaves remain in the plants (23–27) had no significant influence on low-potassium content of fruit and yield. However, if 23 leaves remain in the plant fruit potassium can be decreased to about 27%. Therefore, our results demonstrate that 50% standard nutrient supply during vegetative growth to anthesis and potassium nutrition restriction after 1 week of anthesis can produce low-potassium melon in perlite culture. Our further research will focus on the increasing efficiency of melon plants to sink the photosynthetic assimilate in fruits and increase quality especially soluble solids, antioxidants, phenols, carotenoids, and other human nutrition related qualities.

## Author Contributions

MA and TA designing and performing the experiments. MA and MT yield and growth measurement. MA, MT, and HT atomic absorption analysis of fruits and nutrient samples. HT, MU, MK, SY, TB, and TA quality testing and clinical validation. MA paper preparation. TA research coordination.

## Conflict of Interest Statement

The authors declare that the research was conducted in the absence of any commercial or financial relationships that could be construed as a potential conflict of interest. The reviewer AC and handling Editor declared their shared affiliation at the time of the review.
