# Supplementary material for: Production of Low-Potassium Content Melon Through Hydroponic Nutrient Management Using Perlite Substrate
Source: Front Plant Sci. 2018 Sep 19;9:1382. doi: 10.3389/fpls.2018.01382 (PMC6157450; doi:10.3389/fpls.2018.01382)
Supplement: Supplementary file 1 [file Table_1.docx]

**Supplementary Table S1.** Full strength “Enshi” nutrient solution

| Chemicals | Amounts^z^ (g/1000 L) |
| --- | --- |
| Ca(NO_3_)_2_.4H_2_O | 950 |
| KNO_3_ | 810 |
| MgSO_4_.7H_2_O | 500 |
| NH_4_H_2_PO_4_ | 155 |
| H_3_BO_3_ | 3 |
| ZnSO_4_.7H_2_O | 0.22 |
| MnSO_4_ .4H2O | 2 |
| CuSO_4_ .5H_2_O | 0.05 |
| Na_2_MoO_4_ .2H_2_O | 0.02 |
| NaFe-EDTA | 25 |

^z^Amounts of salts per 1000 L of tap water (Hori,1966).
