# Supplementary material for: Production of Low-Potassium Content Melon Through Hydroponic Nutrient Management Using Perlite Substrate
Source: Front Plant Sci. 2018 Sep 19;9:1382. doi: 10.3389/fpls.2018.01382 (PMC6157450; doi:10.3389/fpls.2018.01382)
Supplement: Supplementary file 2 [file Table_2.docx]

**Supplementary Table S2.** Mineral nutrient content of tap water used for this study. Measurements were conducted at 25 ºC.

| EC (dS/m) | pH | Mineral nutrients (ppm) | | | | | | |
| --- | --- | --- | --- | --- | --- | --- | --- | --- |
|  |  | NO_3_^-^-N | PO_4_^3-^ | K^+^ | Ca^2+^ | Mg^2+^ | Fe^3+^ | Na^+^ |
| 0.22 | 7.9 | 18.7 | 2.1 | 1.0 | 19.9 | 7.1 | 0.01 | 18.5 |
