# Supplementary material for: Production of Low-Potassium Content Melon Through Hydroponic Nutrient Management Using Perlite Substrate
Source: Front Plant Sci. 2018 Sep 19;9:1382. doi: 10.3389/fpls.2018.01382 (PMC6157450; doi:10.3389/fpls.2018.01382)
Supplement: Supplementary file 3 [file Table_3.docx]

**Supplementary Table S3.** Nutrient solution supply schedule for melon grown in hydroponics (10 plants) and in perlite substrate (60 plants) (Experiment I)

| **Vegetative growth stages (weeks)** | | | | **Reproductive growth stages (weeks)** | | | | | | |
| --- | --- | --- | --- | --- | --- | --- | --- | --- | --- | --- |
| 1^st^ | 2^nd^ | 3^rd^ | 4^th^ | 5th | 6^th^ | 7th | 8th | 9th | 10^th^ | 11^th^ |
| 50% standard nutrient solution | | * | | 50% standard nutrient solution without KNO_3_ till harvest. | | | | | | |

*Melon plants were supplied with KNO_3_ in four splits viz. 50, 75, 100 and 125% of required KNO_3_ (Asao et al., 2013);

*The amount of KNO_3_ required per plant was calculated in hydroponic culture of melon;

*Plants grown with 50% standard nutrient solution in perlite and in hydroponics were used as control.
