# Supplementary material for: Production of Low-Potassium Content Melon Through Hydroponic Nutrient Management Using Perlite Substrate
Source: Front Plant Sci. 2018 Sep 19;9:1382. doi: 10.3389/fpls.2018.01382 (PMC6157450; doi:10.3389/fpls.2018.01382)
Supplement: Supplementary file 4 [file Table_4.docx]

**Supplementary Table S4.** Nutrient solution supply schedule for 8 weeks of culture of 60 melon plants (Experiment II)

| **K supply (ml)** | **Dates (month/day)** | | | | | | | |
| --- | --- | --- | --- | --- | --- | --- | --- | --- |
|  | **8/11** | **8/13** | **9/8** | **9/15** | **10/13** | **10/16** | **10/20** | **11/3** |
| Feeding solution (ml/ week) | 540 | 624 | 24336 | 5460 | 39312 | 3510 | 3744 | 9828 |
| Concentration (%) | 50 | 50 | 50 | 50 | 50 | 50 | 50 | 50 |
| Total supply (ml) | 540 | 1144 | 25480 | 30940 | 70252 | 73762 | 77506 | 87334 |
