# Supplementary material for: Production of Low-Potassium Content Melon Through Hydroponic Nutrient Management Using Perlite Substrate
Source: Front Plant Sci. 2018 Sep 19;9:1382. doi: 10.3389/fpls.2018.01382 (PMC6157450; doi:10.3389/fpls.2018.01382)
Supplement: Supplementary file 5 [file Table_5.docx]

**Supplementary Table S5.** Nutrient solution supply schedule for 175 melon plants (Experiment III)

| **Nutrient solution (ml)** | **Dates (month/day)** | | | | | | | | | | |
| --- | --- | --- | --- | --- | --- | --- | --- | --- | --- | --- | --- |
|  | **5/3** | **5/15** | **5/20** | **5/22** | **5/28** | **6/4** | **6/12** | **7/17** | **7/24** | **7/27** | **7/30** |
| Feeding solution (ml/times) | 250 | 150 | 200 | 200 | 250 | 300 | 350 | 300 | 200 | 150 | 100 |
| Times/day | 6 | 11 | 11 | 13 | 13 | 13 | 13 | 13 | 13 | 13 | 13 |
| Concentration (%) | 50 | 50 | 50 | 50 | 75 | 75 | 75 | 75 | 75 | 75 | 75 |
| Culture solution (ml/day) | 1500 | 1650 | 2200 | 2600 | 3250 | 3900 | 4550 | 3900 | 2600 | 1950 | 1300 |
