# Supplementary material for: Production of Low-Potassium Content Melon Through Hydroponic Nutrient Management Using Perlite Substrate
Source: Front Plant Sci. 2018 Sep 19;9:1382. doi: 10.3389/fpls.2018.01382 (PMC6157450; doi:10.3389/fpls.2018.01382)
Supplement: Supplementary file 6 [file Table_6.docx]

**Supplementary Table S6.** Nutrient solution supply schedule for 175 melon plants (Experiment IV)

| **Nutrient solution (ml)** | **Dates (month/day)** | | | | | | | | | | | |
| --- | --- | --- | --- | --- | --- | --- | --- | --- | --- | --- | --- | --- |
|  | **4/11** | **4/13** | **5/3** | **5/14** | **5/20** | **5/22** | **6/4** | **6/18** | **7/4** | **7/8** | **7/11** | **7/13** |
| Feeding solution (ml/times) | 150 | 225 | 300 | 225 | 300 | 300 | 375 | 450 | 350 | 250 | 150 | 50 |
| Times/day | 6 | 6 | 6 | 11 | 12 | 13 | 13 | 13 | 13 | 13 | 13 | 13 |
| Concentration (%) | 50 | 50 | 50 | 75 | 75 | 75 | 75 | 75 | 75 | 75 | 75 | 75 |
| Culture solution (ml/day) | 900 | 1350 | 1800 | 2475 | 3600 | 3900 | 4875 | 5850 | 4550 | 3250 | 1950 | 650 |
